# EEG Features of Cortical Intent for Urination and Defecation in Healthy Aging and Spinal Cord Injury: A Descriptive Feasibility Study for BCI Neurorehabilitation

**DOI:** 10.3390/brainsci16101126

**Published:** 2026-10-18

**Authors:** Chuan Xue, Xiangrui Cai, Ziqi Guo, Haoran Xu, Jiale Xie, Rui Ji, Lei Cao, Jie Jia

**Affiliations:** 1 Department of Rehabilitation Medicine, Huashan Hospital, Fudan University, Shanghai 200040, China; xuechuan16@163.com (C.X.); guoziqi@fudan.edu.cn (Z.G.); oranrehab@163.com (H.X.); 13785492133@163.com (R.J.); 2 Department of Artificial Intelligence, Shanghai Maritime University, Shanghai 200120, China; caixiangrui_bci@163.com (X.C.); lcao@shmtu.edu.cn (L.C.); 3 The First Affiliated Hospital of Fujian Medical University, Fuzhou 350005, China; xiejiale99@163.com; 4 National Clinical Research Center for Aging and Medicine, Huashan Hospital, Fudan University, Shanghai 200040, China

**Keywords:** spinal cord injury, brain–computer interface, electroencephalography (EEG), neurorehabilitation, event-related desynchronization, functional connectivity, neurogenic lower urinary tract dysfunction (NLUTD), neurogenic bowel dysfunction (NBD)

## Abstract

**Highlights:**

**What are the main findings?**

- The sensorimotor cortex exhibits consistent broadband event-related desynchronization (ERD) during visceral motor attempt, while the frontal cortex shows marked frequency-specific differentiation.
- Healthy older adults showed significantly greater SMC beta2 ERD during suppressed urination and defecation motor attempts, while SCI patients showed a non-significant trend.

**What are the implications of the main findings?**

- High-level cortical motor intentions for bowel and bladder control appear to be stable despite peripheral neural conduction blockades.
- These spatiotemporal EEG signatures may provide a potential neurophysiological basis for developing targeted, closed-loop brain–computer interfaces for pelvic neurorehabilitation.

**Abstract:**

**Background/Objectives**: Systematically elucidating the neural mechanisms of bowel and bladder control is crucial for developing closed-loop brain–computer interface (BCI) neurorehabilitation for patients with spinal cord injury (SCI). This study aims to characterize the spatiotemporal Electroencephalography (EEG) dynamics and compensatory cortical reorganization underlying continence motor intentions across healthy aging and SCI cohorts. **Methods**: 32-channel EEG signals were collected from healthy young adults, healthy older adults, and SCI patients during motor attempt paradigms of suppressed urination and defecation (SUD) and active urination and defecation (UD). Data were analyzed utilizing event-related spectral perturbation (ERSP) and phase-locking value (PLV) functional connectivity across alpha (8–13 Hz), beta1 (13–20 Hz), and beta2 (20–30 Hz) bands. **Results**: All cohorts exhibited generally consistent broadband event-related desynchronization (ERD) in the sensorimotor cortex (SMC) during visceral motor attempt. Conversely, the frontal cortex (FC) demonstrated marked frequency-band differentiation. During the SUD task, healthy older adults showed significantly greater SMC beta2 ERD than young adults, whereas SCI patients showed a similar but non-significant trend. Frontal beta2 findings were preliminary and should be interpreted with caution due to potential residual artifacts. Network analysis revealed that physiological aging and SCI are associated with large-scale topological reconfigurations, prominently featuring enhanced long-range frontal functional connectivity to compensate for sensory or peripheral deficits. **Conclusions**: High-level cortical intentions for continence are preserved after SCI, with frontal high-frequency activity possibly reflecting a compensatory process for sensorimotor disruption. These findings substantiate that visceral intentions exhibit discernible characteristics at the cortical level, establishing a spatiotemporal framework for targeted pelvic nerve modulation via brain–machine interfaces.

## 1. Introduction

Urination and defecation are essential physiological functions requiring precise coordination between the central and peripheral nervous systems [1]. Damage to these pathways—due to spinal cord injury (SCI), stroke, Parkinson’s disease, or multiple sclerosis—often leads to neurogenic lower urinary tract dysfunction (NLUTD) and neurogenic bowel dysfunction (NBD), with a combined prevalence of fecal incontinence and constipation reaching approximately 70% and a substantial negative impact on quality of life [2,3,4,5]. Aging further alters central and peripheral regulatory pathways, pelvic floor neuromuscular control, and bladder function, producing baseline differences in continence regulation across age groups [6]. Current conservative treatments—pelvic floor training, biofeedback, dietary optimization, and peripheral nerve stimulation—remain largely open-loop and passive, and they often fail to engage the cortical top-down control intentions governing bowel and bladder function [7,8,9]. Consequently, their capacity to promote active neural remodeling of injured central pathways is limited, and restoring autonomous continence control remains a major unresolved challenge in neurorehabilitation.

Brain–computer interface (BCI) technology offers a novel active closed-loop paradigm for assessing and rehabilitating functional deficits after central nervous system injury [10]. BCI systems decode neural signals related to motor intention or cognitive tasks and provide contingent feedback, aiming to promote spontaneous recovery by stimulating remodeling of impaired central pathways. In rehabilitation BCIs, motor imagery (MI) and motor attempt (MA) are commonly used. MA refers to the volitional effort to perform a movement despite loss of peripheral motor control [11,12]; it is more naturalistic than MI and often yields stronger cortical activation and better decoding accuracy [13,14]. Based on Hebbian plasticity, real-time decoding of cortical intention combined with synchronous peripheral afferent modulation within the intention window may recreate the synaptic coincidence required for plasticity and help reconstitute volitional control circuits [15]. However, rehabilitation BCIs have predominantly targeted limb motor function; investigations of central electrophysiological representations underlying complex pelvic visceral tasks such as micturition and defecation remain scarce.

Prior functional MRI studies indicate that pelvic visceral control is primarily localized to the sensorimotor cortex (SMC) and frontal cortical areas (FC), which operate in coordination [16,17]. In the EEG domain, Colombo et al. analyzed power spectra during micturition and identified cortical regions involved in inhibition and release phases, while noting the methodological challenge of muscle and movement artifacts [18]. More recently, Zhou et al. demonstrated the feasibility of using wearable single-channel EEG to detect urinary urgency and voiding intention with deep learning [19,20]. These studies suggest that urination and defecation are governed by distributed cortical networks, providing a spatial foundation for EEG-based investigation. SCI disrupts sensorimotor pathways and induces extensive cortical reorganization. Recent work has shown that MI-based BCI combined with robotic gait training can improve cortical μ and β rhythm modulation and bladder function in SCI individuals, with frontal and parietal β-band activity correlating with symptom improvement [21,22]. The phase-locking value (PLV) has been widely used to quantify frequency-specific functional connectivity [23], but its application to pelvic visceral motor control in aging and SCI remains largely unexplored. Normal aging is associated with more widespread and bilateral cortical activation, altered alpha-mu and beta oscillations, and changes in motor network connectivity, which may also affect visceral motor control [24,25,26,27,28,29].

Despite this progress, the cortical activation patterns and electrophysiological features of human bowel and bladder control remain poorly understood, hindering the transition from mechanism discovery to machine recognition. Drawing on central nervous system plasticity and Hebbian theory, this study proposes the “cortical recognizability hypothesis of bowel and bladder control intent”: although SCI disrupts the physical neural conduction pathways from the central nervous system to pelvic effectors, the cortical top-level control intention regulating micturition and defecation retains specific electrophysiological signatures with frequency-band and spatial specificity across subjects varying in age, disease duration, and type of neural injury. To test this hypothesis, we developed a standardized EEG neurofeedback and motor-attempt paradigm for SCI patients and healthy individuals. We compared wireless multichannel EEG signals during active suppression of urination and defecation (SUD) and active urination and defecation attempts (UD) in three cohorts: healthy young adults (age < 60), healthy older adults (age > 60), and SCI patients with bladder/bowel dysfunction. Event-related spectral perturbation (ERSP) values were computed in alpha (8–13 Hz), beta1 (13–20 Hz), and beta2 (20–30 Hz) bands, and whole-brain PLV functional connectivity was analyzed. This study aims to characterize the frequency specificity, spatial distribution, and temporal dynamics of bladder/bowel motor intention across groups, thereby filling a gap in the electrophysiological characterization of human pelvic visceral control and providing a foundation for targeted BCI-based neuromodulation.

## 2. Materials and Methods

### 2.1. Subjects and Ethics

#### 2.1.1. Recruitment and Grouping of Clinical Samples

This study recruited four representative participant groups from Huashan Hospital, Fudan University, the Shandong Provincial Third Hospital, and Ninghai Second Hospital: a healthy young control group, a healthy older control group, and a spinal cord injury patient group.

#### 2.1.2. Patients with Spinal Cord Injury

This study enrolled patients with SCI who were seen between June 2024 and April 2025 at Huashan Hospital affiliated with Fudan University, Ninghai County Second Hospital, and the Third Provincial Hospital of Shandong. Detailed clinical characteristics of patients with spinal cord injury are presented in Supplementary Table S1. Inclusion criteria: age 18–75 years, any sex; patients with impaired or absent voluntary bowel or bladder control due to spinal cord injury; suprasacral spinal cord lesions; American Spinal Injury Association (ASIA) impairment scale grades A, B, C, or D, with no restriction on time since onset; no significant cognitive impairment (Mini-Mental State Examination: illiterate > 17, primary school > 20, junior high school and above >24); and provision of written informed consent. Exclusion criteria: scalp or skin surface lesions; intracranial metallic implants; unilateral spatial neglect or visual field deficits; pregnant or lactating women; unstable angina, coronary stent implantation within the past six months, or cardiac pacemaker implantation; any other condition that the investigator deems unsuitable for participation in the trial; participation in or within three months prior to consent having participated in another clinical trial. Withdrawal criteria: subjects who are unwilling or unable to continue participation; discovery after enrollment that the subject does not meet inclusion criteria or meets any exclusion criterion; occurrence of intolerable adverse events or serious adverse events such that the investigator judges that continued participation would impose risks to the subject that outweigh potential benefits.

#### 2.1.3. Healthy Control Group

This study enrolled healthy volunteers who attended Huashan Hospital affiliated with Fudan University, Ninghai County Second Hospital, and Shandong Provincial Third Hospital between June 2024 and April 2025. Inclusion criteria: no history of central or peripheral nervous system disease; age 18–75 years, any sex; absence of significant cognitive impairment; provided informed consent and voluntarily agreed to participate. Exclusion criteria: scalp or skin surface lesions; intracranial metal implants; hemispatial neglect or hemianopia; pregnant or lactating women; unstable angina, coronary stent implantation within the past six months, or cardiac pacemaker implantation; any other conditions judged by the investigators to render participation inappropriate; participation in any other clinical trial within three months prior to signing the informed consent or ongoing participation in such a trial. Discontinuation criteria: subject unwilling or unable to continue participation; discovery after enrollment that the subject does not meet inclusion criteria or meets any exclusion criterion; occurrence of intolerable adverse events or serious adverse events, or investigator judgment that the risks of continued participation outweigh potential benefits.

#### 2.1.4. Ethics Approval and Registration Statement

This study protocol strictly adheres to the Declaration of Helsinki and has been reviewed and approved by the institutional ethics committees of all participating centers, including: the Ethics Committee of Huashan Hospital affiliated with Fudan University (Approval No.: 2025-103), the Medical Ethics Committee of Shandong Provincial Third Hospital (Approval No.: KYLL-2024099), and the Ethics Committee of Ninghai County Second Hospital (Approval No.: SL2024-14). This study has been formally registered with the Chinese Clinical Trial Registry under registration number ChiCTR2500102761.

### 2.2. Experimental Design and BCI Paradigm

To effectively extract subjects’ high-level central control intentions governing the brain regulation of complex visceral bowel and bladder movements, this study established a standardized paradigm for collecting electroencephalographic neurofeedback and motor attempt EEG signals related to defecation and urination. Subjects were seated comfortably in a chair or wheelchair in front of a computer display and emptied their bladder and rectum before donning the EEG cap.

**Figure 1:**
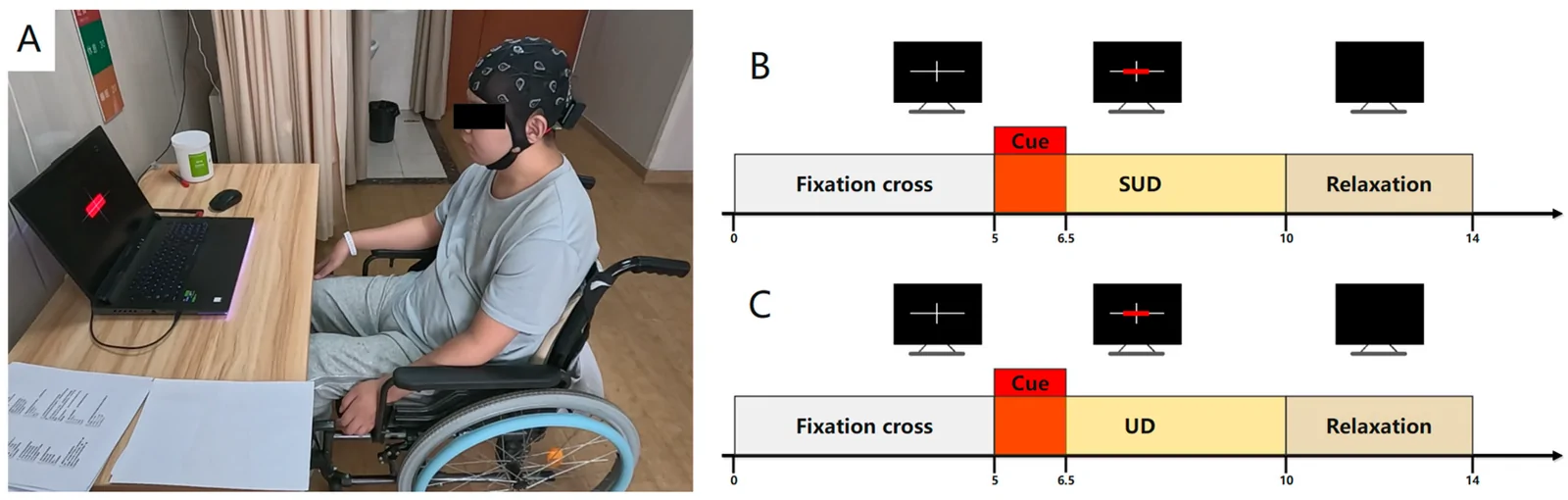
Experimental setup and brain–computer interface (BCI) motor attempt paradigms. (**A**) Real-world setting of the EEG signal acquisition, where subjects were seated in a wheelchair in front of a computer display wearing an EEG cap. (**B**) Schematic of the Suppressed Urination and Defecation (SUD) task paradigm. (**C**) Schematic of the active Urination and Defecation (UD) task paradigm.

The SUD and UD tasks share an identical trial structure. At T = 0 s, a cross appears to indicate the start of a trial. As shown in Figure 1 and Videos S1–S3, at T = 5 s, upon presentation of a red square cue, participants are instructed to initiate a 5 s motor attempt, which terminates when the cross disappears. SUD Task: Participants attempt a pelvic floor contraction (urine- and feces-retention effort) by performing an isometric contraction to the greatest extent possible. UD Task: Participants attempt a voiding (urination/defecation) movement while avoiding a Valsalva maneuver as much as possible. For both tasks, the experiment comprises a single run of 40 trials with a 4 s inter-trial interval, lasting approximately 10 min. Participants are required to maintain visual fixation on the anterior display throughout each trial.

### 2.3. Electroencephalographic Signal Acquisition

Multichannel spontaneous scalp EEG signals were recorded in real time using a 32-channel wireless high-precision EEG/ERP acquisition system (iRecorderW32, Shanghai Niantong Intelligent Co., Ltd. Shanghai, China). The 32 recording electrode locations were arranged strictly in accordance with the international 10–20 electrode placement system (including Fp1, Fp2, AF3, AF4, Fz, F3, F4, F7, F8, FC1, FC2, FC5, FC6, Cz, C3, C4, T7, T8, CP1, CP2, CP5, CP6, Pz, P3, P4, P7, P8, PO3, PO4, Oz, O1, O2). The online physical reference electrode was placed at FCz, and the ground electrode was positioned at AFz on the forehead. The system sampling rate was set to 500 Hz online. During the experiment, a dedicated conductive gel was applied to the scalp and the impedance of all scalp electrodes was monitored in real time and maintained below 30 kΩ.

### 2.4. Offline Preprocessing of Electroencephalographic Signals

Offline EEG data preprocessing was conducted in the MATLAB (R2022b) environment using the advanced EEG analysis toolbox Letswave7. Digital filtering employed a fifth-order Butterworth zero-phase digital filter to perform a 0.5–30 Hz bandpass on the raw EEG signals, and a cascaded 50 Hz notch filter was applied to suppress mains power-line interference. Bad channels were identified by visual inspection or variance thresholds and interpolated using 3D spherical spline spatial interpolation, followed by re-referencing to a common average reference (Common Average Reference, CAR). Ocular and physiological artifacts were removed by running extended independent component analysis (ICA), accurately identifying and removing vertical and horizontal eye-movement components based on their scalp topographies and temporal characteristics, thereby maximizing recovery of fine cortical components related to visceral movement. Long epochs were extracted using the task cue onset as time zero, yielding segments from −3 s to 9 s.

### 2.5. Region of Interest (ROI) Definition for Brain Function

**Figure 2:**
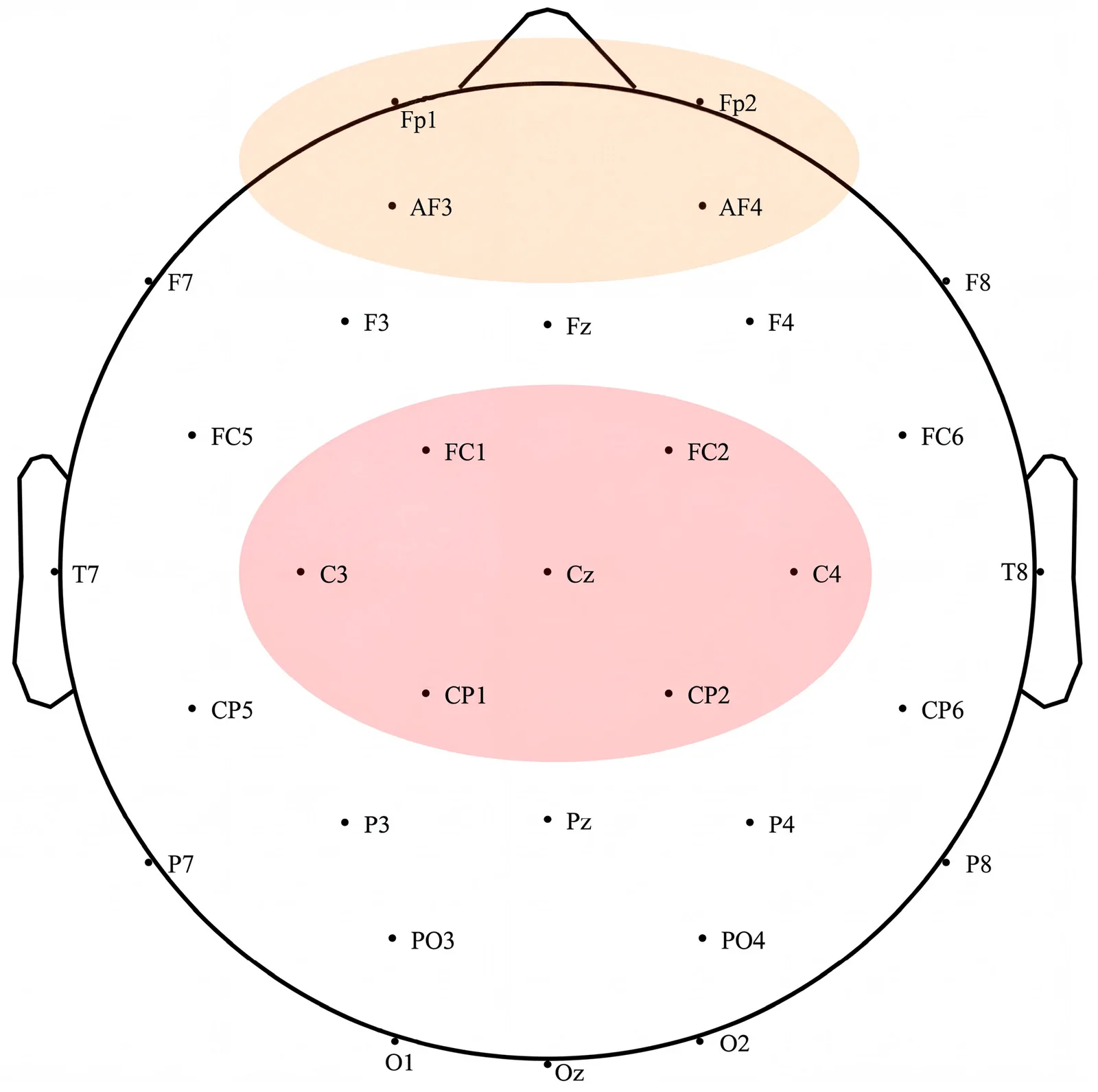
Schematic of EEG electrode selection for motor-related cortical regions. Note: Red regions denote sensorimotor areas; selected electrodes include FC1, FC2, Cz, C3, C4, CP1, CP2. Orange regions denote frontal cortical areas; selected electrodes include Fp1, Fp2, AF3, AF4.

Previous functional MRI studies indicate that cortical regions responsible for regulating defecation and micturition movements are primarily localized to the SMC and FC areas [16,17]. In the present study, FC1, FC2, Cz, C3, C4, CP1, and CP2 were selected as SMC electrodes, while Fp1, Fp2, AF3, and AF4 were designated as FC electrodes (Figure 2). Consistent with prior work [30,31,32], averaging multiple trials from 12–15 subjects allows clear observation at the individual level of movement-related event-related desynchronization/event-related synchronization (ERD/ERS) associated with movement preparation and execution, which satisfies the basic requirements for statistical power in inferential testing.

To investigate differences in the temporal evolution of spectral power in the Alpha, β1, and β2 bands between SUD and UD tasks, this study first generated ERSP time–frequency maps based on 11 key electrodes. To further characterize task responses in the SMC and FC, time–frequency analyses were performed on representative electrodes for the SMC (Cz, C3, C4) and the FC (Fp1, Fp2). Building on these analyses, we quantified differences in the temporal distribution of ERD across multiple frequency bands between the movement initiation and maintenance states of the bimanual motor task, and conducted statistical tests on the Alpha, β1, and β2 bands for the SMC (including FC1, FC2, Cz, C3, C4, CP1, CP2 electrodes) and on the β2 band for the FC (including Fp1, Fp2, AF3, AF4 electrodes).

### 2.6. Event-Related Spectral Perturbation Time–Frequency Quantification

To examine power changes in event-related EEG signals in the time and frequency domains, this study computed ERSP. This method effectively characterizes details of ERD and ERS induced by motor execution tasks (in EEG topographies and time–frequency plots, red denotes ERS and blue denotes ERD; negative ERSP values correspond to ERD, positive values to ERS). The ERSP is computed as follows:

(1) $$\mathrm{ERSP}\left(f,t\right)=\frac{1}{n}\sum_{k=1}^{n}{\left(F_{k}(f,t\right)}^{2})$$

Here, *n* denotes the number of trials, and ${F_{k}(f,t)}^{2}$ represents the spectral estimate at frequency f and time t for the k-th trial. ERD refers to a reduction in event-related EEG power relative to spontaneous EEG power during the baseline period. To quantify the ERD pattern, a baseline correction of the ERSP was performed using the interval from −2 to −0.5 s prior to task onset.

To visually present the spatial patterns of ERD during the SUD and UD tasks, mean ERSP values were extracted for the intervals 0–2 s and 2–5 s after task onset, and scalp topographies were plotted for the alpha (8–13 Hz), beta1 (13–20 Hz), and beta2 (20–30 Hz) bands. To reveal the temporal and spectral dynamics of cortical power in specific regions under different tasks, key electrode data were selected and time–frequency representations were plotted based on ERSP values from −2 to 9 s relative to task onset.

### 2.7. Cortical Space Functional Connectivity Analysis

Phase-locking value (PLV) matrices were first calculated separately for each participant under the resting-state and motor-attempt conditions. After excluding five channels that were not included in the analysis, 27 EEG channels remained, yielding 351 unique undirected connections in each network. Within each group-by-task-by-frequency-band combination, subject-level paired-samples t-tests were then performed for all 351 connections. The resulting 351 *p* values within each network were corrected using the Benjamini–Hochberg false discovery rate (BH-FDR) procedure, with statistical significance defined as q < 0.05. Red connections indicate significantly increased PLV during the motor-attempt condition relative to the resting-state condition, whereas blue connections indicate significantly decreased PLV.2.8 Statistical analysis

All hypothesis testing and statistical inference procedures in this section were conducted within offline statistical software environments (SPSS 20 and MATLAB R2022b). Quantitative data were assessed for normality (Q–Q plot or Shapiro–Wilk test) and homogeneity of variance; EEG-derived quantitative metrics conforming to a normal distribution are reported as mean ± standard deviation (x ± s). To ensure robustness of signal analyses, an outlier-preprocessing step was applied prior to statistical inference, whereby extreme spectral power values exceeding three standard deviations from the mean within each group and channel were excluded. A two-tailed significance threshold of *p* < 0.05 was adopted throughout. For evaluation of large-scale whole-brain PLV functional connectivity networks, paired-sample t-tests were used to assess the significance of connectivity strength (PLV values) for each channel pair between the “motor attempt” and “resting baseline” conditions across all participants. To investigate effects of healthy aging and spinal cord injury on central neural representations of bladder–bowel control, one-way ANOVA was employed to test group-level spatial differences in ERSP means within specific frequency bands and core brain regions across three cohorts (young healthy, elderly healthy, and SCI patients). When ANOVA revealed a significant main effect, post hoc multiple comparisons (Tukey HSD or Games–Howell, depending on variance homogeneity) were performed to identify and compare pairwise group differences. One primary hypothesis was pre-specified: during the SUD task, SMC beta2 (20–30 Hz) ERD would differ among groups, with planned contrasts comparing healthy older adults and SCI patients to healthy young adults. The primary family consisted of one omnibus one-way ANOVA and two planned contrasts. The two planned contrast *p* values were adjusted using the Holm–Bonferroni procedure. All remaining ERSP analyses were classified as secondary/exploratory and were corrected using the Benjamini–Hochberg false discovery rate (BH-FDR) across the pre-defined family of the remaining 11 task × ROI × band tests. Within-region frequency-band comparisons were corrected using BH-FDR across the 12 group × task × ROI tests. LSD post hoc comparisons were not used. PLV analyses were corrected using BH-FDR within each group × task × band family of 351 edges. A two-tailed threshold of q < 0.05 was used for multiplicity-controlled analyses.

## 3. Results

### 3.1. Electroencephalographic Topographical and Time–Frequency Maps of SUD and UD Motor Attempt Tasks in Healthy Subjects and Individuals with Spinal Cord Injury

**Figure 3:**
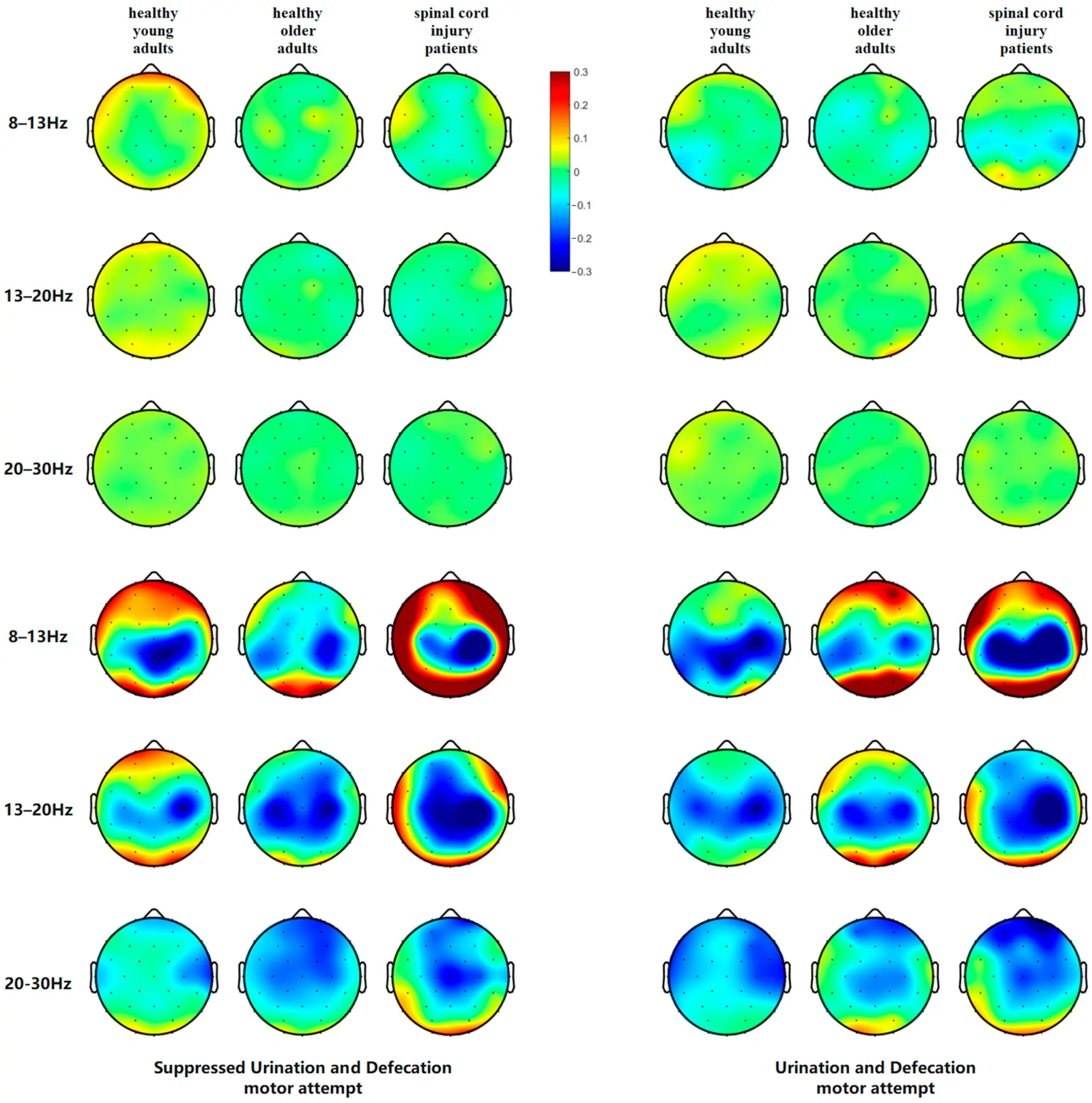
Scalp EEG topographies of healthy subjects and spinal cord injury patients during resting state and bowel/bladder movement attempt tasks. Negative ERSP values indicate event-related desynchronization (ERD; blue regions in the topographies), positive values indicate event-related synchronization (ERS; red regions in the topographies), and values near zero indicate no significant change in regional power (yellow–green regions in the topographies). The top three rows show resting-state EEG topographies; the bottom three rows show EEG topographies during the Suppressed Urination/Defecation and Urination/Defecation attempt tasks.

**Figure 4:**
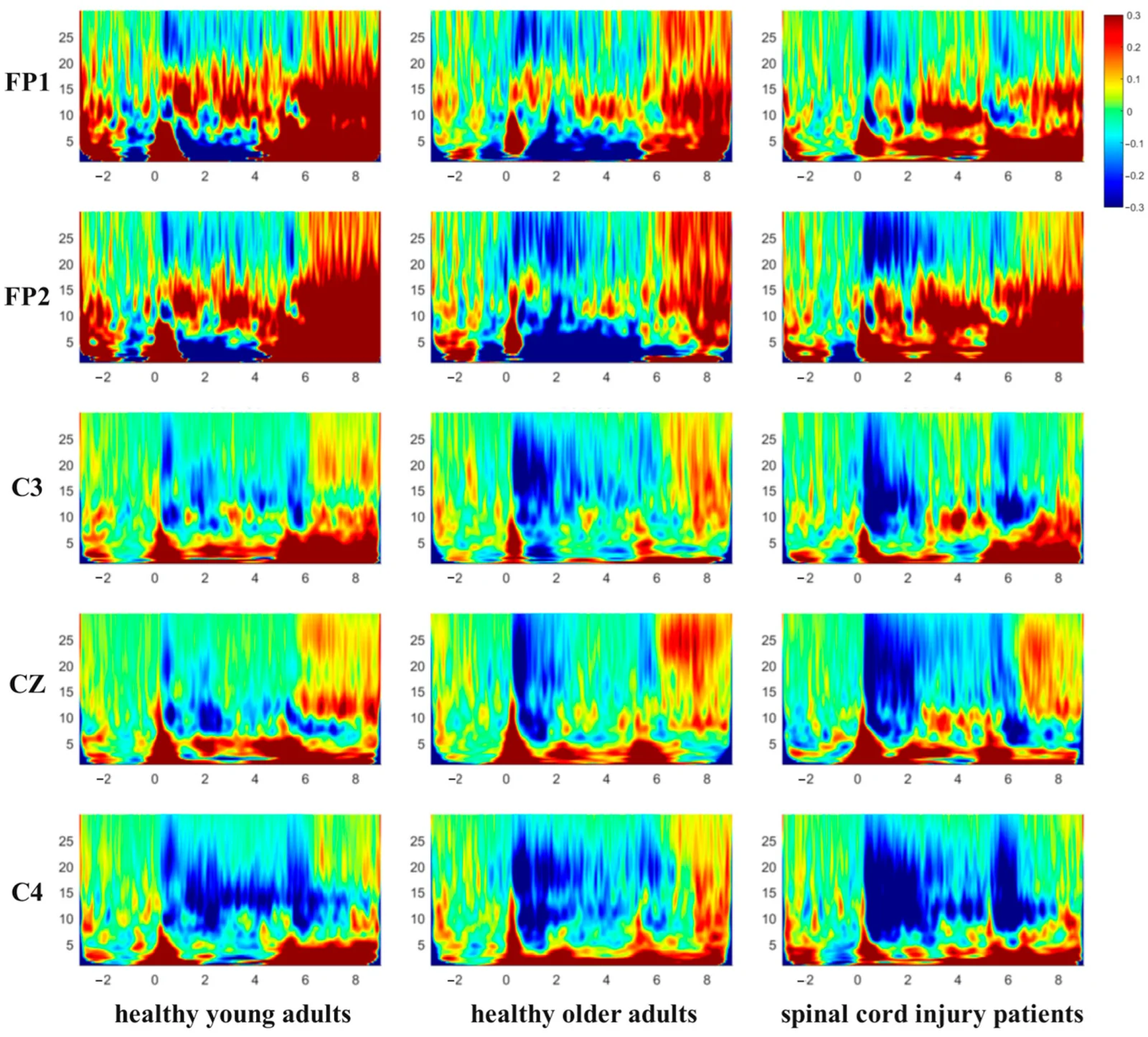
Time–frequency EEG maps of healthy subjects and spinal cord injury patients during suppressed urination and defecation attempt tasks. Note: The horizontal axis denotes the timing of the retention task (seconds); −3 to 0 s represents resting state, 0 to 5 s represents the retention attempt task state, and 5 to 9 s represents the recovery/rest state. The vertical axis denotes EEG frequency (Hz).

**Figure 5:**
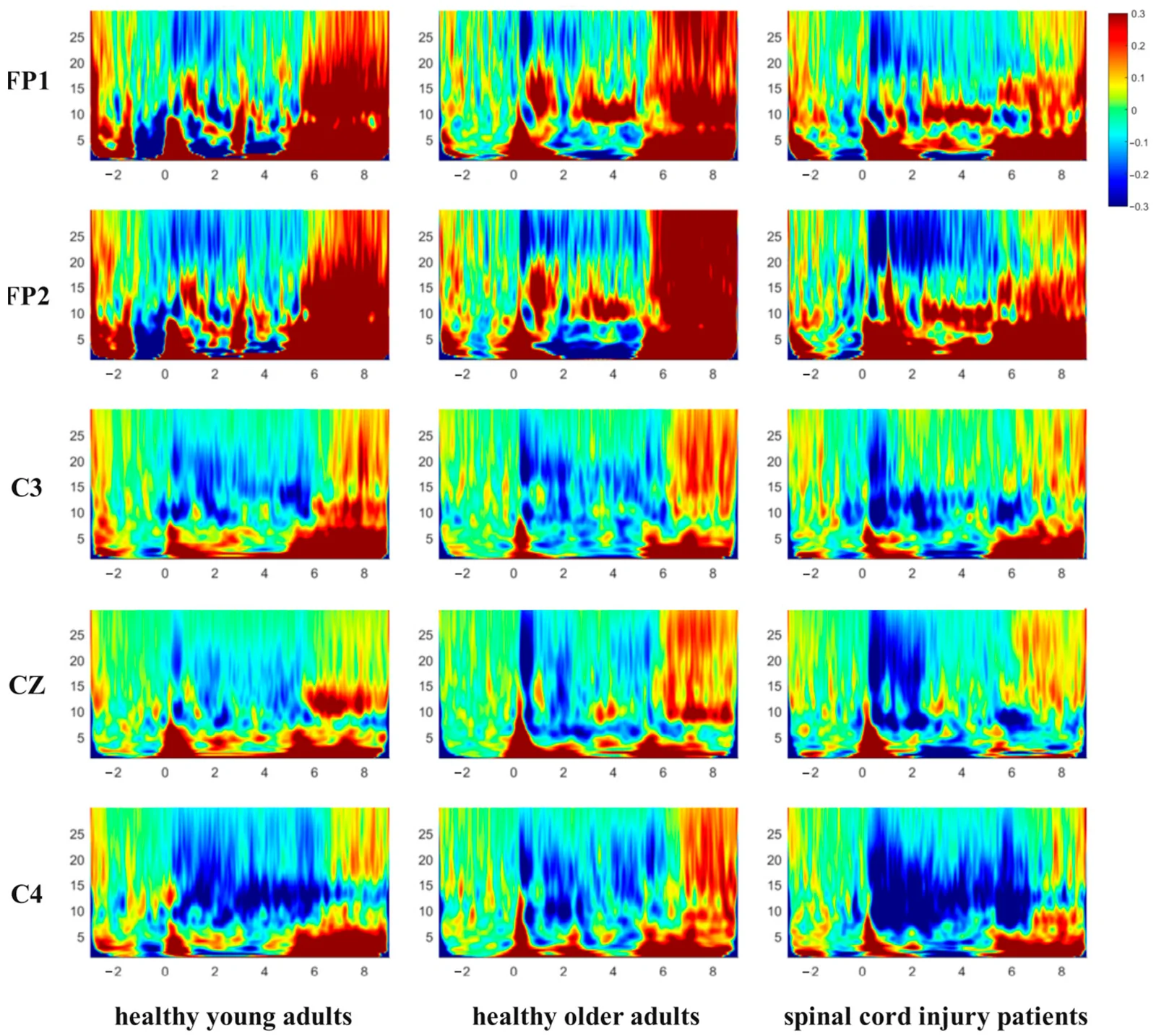
Time–frequency maps of EEG for healthy subjects and spinal cord injury patients during urination and defecation tasks. Note: The horizontal axis represents the time of the urinary and defecatory movement task (seconds), with −3 to 0 s denoting the resting state, 0 to 5 s denoting the urinary/defecatory attempt task state, and 5 to 9 s denoting the recovery/rest state; the vertical axis represents EEG frequency (Hz).

This study recruited a total of 90 participants, comprising 30 healthy young adults, 31 healthy older adults, and 29 individuals with spinal cord injury. To further elucidate the spatiotemporal dynamics of the brain during the execution of complex visceral neural control, this study mapped whole-brain energy topographic maps (Figure 3) and representative channel ERSP time–frequency plots (Figure 4 and Figure 5) for healthy subjects and spinal cord injury patients during SUD and UD tasks. The results indicate that cortical activation patterns underlying bowel and bladder motor control exhibit pronounced frequency-band specificity. Given the clinical heterogeneity of the SCI cohort, we also generated exploratory subgroup topographies stratified by AIS grade, pelvic sensory/motor status, etiology, and disease duration (Supplementary Figures S1–S4). Because of the small and unbalanced sample sizes in these subgroups, no formal statistical testing was performed. These analyses are therefore descriptive and hypothesis generating only.

#### 3.1.1. Alpha and Beta1 Bands

In the time–frequency evolution, when subjects received the visual cue (0 s) and entered the task state, the lower and mid-frequency bands (Alpha: 8–13 Hz and Beta1: 13–20 Hz) in the SMC exhibited pronounced and sustained ERD (depicted as deep blue patches in the figure).

On the spatial-topological distribution of whole-brain topographies, significant ERD energy in the alpha and Beta1 bands was highly concentrated in the midline and bilateral centro-parietal regions. This spatial characteristic indicates that during the execution of SUD and UD movement attempts, in both healthy cohorts and SCI patients, the issuance of primary motor intent and the interaction with skeletal muscle and visceral proprioception are associated with activation of the parietal motor control network. Given the peripheral execution constraints in SCI patients, this pronounced parietal SMC activation further corroborates that the movement-attempt paradigm effectively recruits top-down resources of the higher motor cortex.

#### 3.1.2. Beta2 Band

As the analyzed frequency band shifts upward into the Beta-2 range, the spatial activation patterns in EEG topographies and time–frequency maps undergo marked band-specific reconfiguration. Electrophysiological signatures of FC begin to emerge prominently. Topographic maps indicate that neural energy perturbations in the Beta-2 band show a pronounced tendency to shift toward the polar and prefrontal regions. The frontal cortex, as the locus of higher-order cognitive control, allocation of attentional resources, and voluntary inhibition, exhibits enhanced activation at higher frequencies; this suggests that bi-phasic control is not a unitary “initiate–execute” motor sequence but may involve substantial engagement of cognitive networks. In tasks that demand strong suppression of peripheral reflexes, such as those characteristic of SUD, high-frequency frontal activity provides tentative evidence consistent with a possible frontal compensatory role in sustaining stable cognitive intervention and focused intentionality.

Frequency-specific spatiotemporal analysis suggests a dual neural mechanism underlying human continence control: the low-to-mid frequency bands (Alpha, Beta-1) primarily reflect direct motor execution and sensory feedback control concentrated in the parietal cortex, whereas the high-frequency band (Beta2) reveals the frontal cortex’s central involvement in higher-order cognitive control and inhibitory regulation. However, these frontal beta2 results should be interpreted with caution, as Fp1/Fp2/AF3/AF4 are close to the eyes and forehead muscles, and ICA-based correction cannot fully exclude residual EMG contamination in the 20–30 Hz band.

### 3.2. Comparison of EEG Time–Frequency Characteristics

To investigate the process of healthy aging and the impact of spinal cord injury on central nervous system representations of bowel and bladder control, this study employed one-way ANOVA to compare ERSP values in the SMC and FC across the Alpha, Beta1, and Beta2 frequency bands among healthy young adults (*n* = 30), healthy older adults (*n* = 31), and patients with spinal cord injury (*n* = 29). Detailed statistical results are presented in Table 1.

#### 3.2.1. Widespread Cortical Representations of Movement Intent

As shown in Table 1, during both SUD and UD motor attempt tasks, the SMC elicited consistent ERD in the Alpha (8–13 Hz), Beta1 (13–20 Hz), and Beta2 (20–30 Hz) bands across healthy young adults, healthy older adults, and SCI patients. This indicates that, when attempting to initiate or suppress pelvic floor muscle and visceral activity, the SMC region exhibits a reliable ERD in all three populations, suggesting that cortical regulation of higher-order motor intent for continence and the associated electrophysiological activation patterns are preserved despite disruption of descending spinal pathways in SCI patients. Concurrently, in the FC region, Alpha and Beta1 bands predominantly exhibited weak ERS, with only a mild ERD observed in the high-frequency Beta2 band.

#### 3.2.2. Inter-Group Variability Analysis of SUD Tasks

In the SUD task, one-way ANOVA indicated that the three groups differed significantly only in the high-frequency band of the SMC (Beta2, 20–30 Hz) (F = 3.452, *p* = 0.035, gatekeeper). Subsequent multiple comparisons (Games-Howell test) revealed that the ERD amplitude in the Beta2 band for the healthy elderly group (−0.153 ± 0.169) was significantly greater than that of the healthy young group (−0.060 ± 0.125) (*p* = 0.022, Holm–Bonferroni q = 0.044). The SCI group likewise exhibited a trend toward stronger ERD activation in the same band (−0.154 ± 0.213) relative to the young group, but this difference did not reach statistical significance (*p* > 0.05, Holm–Bonferroni q > 0.05). No significant differences among the three groups were observed in other SMC bands or in any FC bands (*p* > 0.05). These results suggest that, during performance of the “hold” movement, which demands substantial cognitive inhibition, healthy aging may elicit greater cortical recruitment of higher-order Beta2-band neural control resources as a compensatory mechanism.

#### 3.2.3. Analysis of Inter-Task Variability in UD

In the UD task, the three cohorts did not exhibit any statistically significant differences across all analyzed frequency bands (Alpha, Beta1, and Beta2) within the SMC and FC regions (all *p* > 0.05). Compared with the young cohort, neither the elderly cohort nor the SCI patients showed significant shifts or degradation in the magnitude of ERD within the core sensorimotor network or in frontal cognitive resource recruitment when executing the “voiding” motor command, suggesting a high degree of task-related neural response homogeneity.

### 3.3. Frequency-Specific Statistical Analysis Within Brain Regions of Healthy Subjects and Spinal Cord Injury Patients

To quantitatively verify the spatial functional specificity of different frequency bands in SUD and UD motor attempt, this study further compared the mean ERSP differences in three frequency bands—Alpha (8–13 Hz), Beta1 (13–20 Hz), and Beta2 (20–30 Hz)—within specific cortical regions (SMC and FC) across three participant groups (healthy young adults, healthy older adults, and spinal cord injury patients) (Table 2), and related these comparisons to the spatial evolution patterns observed in the topographic maps.

#### 3.3.1. Broadband Activation of the Sensorimotor Cortex

One-way ANOVA results indicated that, in both the SUD and UD tasks, the three groups did not exhibit statistically significant differences in the amplitude of ERD in the SMC across the Alpha, Beta1 and Beta2 frequency bands (all *p* > 0.05). As the primary cortical hub for executing and maintaining pelvic floor/visceral movements, the SMC reliably elicits a broadband ERD spanning low-to-high frequencies (8–30 Hz) following initiation commands. This global desynchronization without band-specific differentiation is a characteristic signature of the human brain issuing strong and sustained direct motor intent to peripheral effectors.

#### 3.3.2. Spectral Functional Differentiation of the Frontal Cortex

In sharp contrast to the SMC, the electrophysiological characteristics within the FC exhibited marked frequency-band-specific differentiation. Statistical analysis revealed that in the low-to-mid frequency bands (Alpha and Beta1), the frontal polar and frontal regions generally showed slight ERS; however, when the frequency increased to the Beta2 band (20–30 Hz), a significant reversal toward negative ERD occurred across these sites. This within-frontal “band splitting” phenomenon was particularly pronounced in groups with physiological functional decline and in those with central impairment.

Analysis of variance and post hoc multiple comparisons of the extracted core data reveal the following: In the UD task, the elderly healthy group exhibited highly significant frontal-band differences (*p* = 0.0002), with the high-frequency Beta2 band eliciting a pronounced event-related desynchronization (ERD = −0.175), whereas the Alpha band displayed strong event-related synchronization (ERS = 0.250). The SCI patient group likewise demonstrated a highly significant band divergence within the frontal region during the UD task (*p* = 0.0009), with Beta2-band activation producing an ERD as large as −0.261. In the SUD task, the SCI group showed a band effect that was significant before correction (*p* = 0.020); however, this effect did not survive BH-FDR across the 12 band-effect tests and is therefore reported as an exploratory trend. By contrast, the young healthy group did not show significant within-frontal-band differences under any task condition (*p* > 0.05).

### 3.4. Phase-Locking Value Functional Connectivity Network Characteristics

To investigate the cooperative mechanisms by which the brain regulates attempted bimanual movements at the whole-brain network interaction level, this study computed whole-brain PLV for each participant and systematically categorized the results by population group (healthy young adults, healthy older adults, and spinal cord injury patients) and by frequency bands (Alpha, Beta1, and Beta2). PLV connection strengths were extracted separately for resting state (Rest) and motor attempt state (Motor).

#### 3.4.1. Whole-Brain Functional Connectivity Characteristics of the Healthy Young Cohort

**Figure 6:**
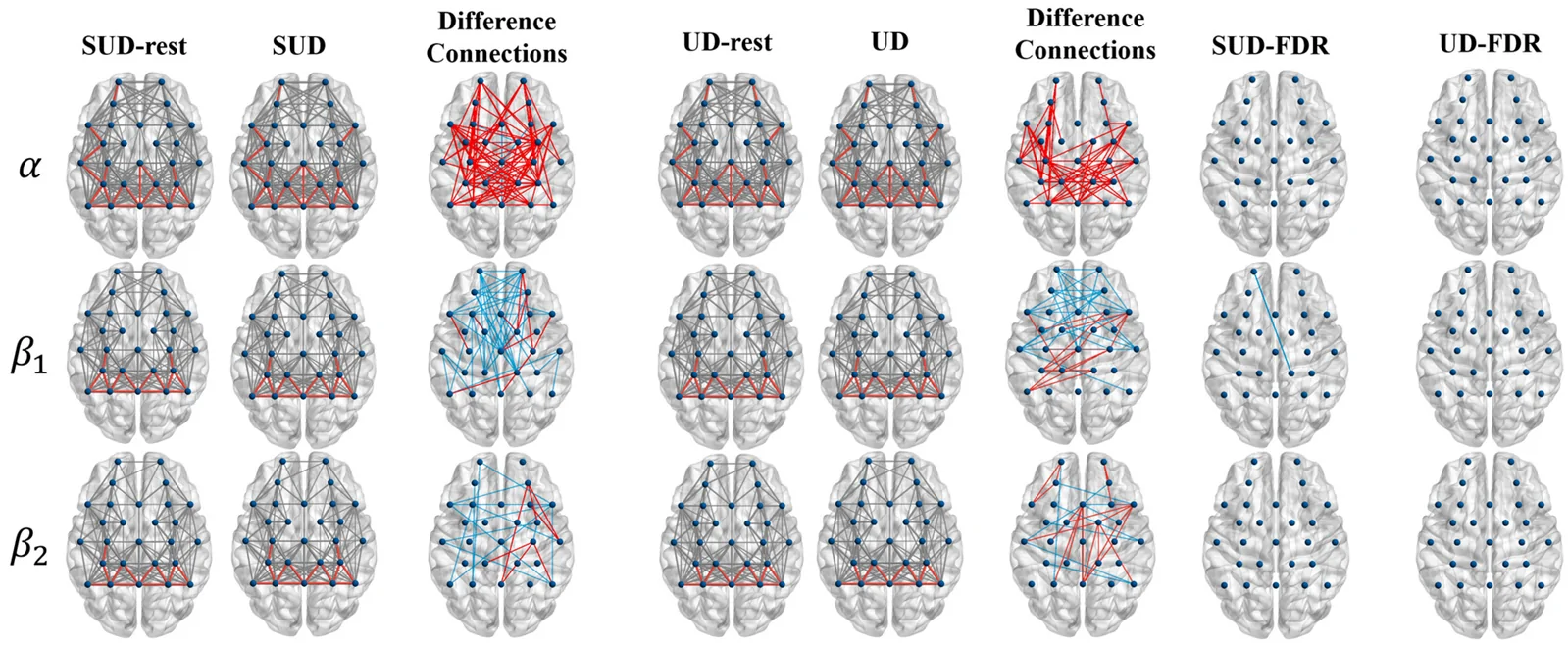
Whole-brain phase-locking value functional connectivity network characteristics in healthy young adults during attempted withholding of urination and defecation. SUD—withholding urination and defecation; UD—voiding and defecation; α—8–13 Hz; β1—13–20 Hz; β2—20–30 Hz; differential connection locations: red indicates increased connectivity; blue indicates decreased connectivity.

Statistical tests indicate that healthy young adults exhibit significant differences in whole-brain PLV functional connectivity across all frequency bands (Alpha, Beta1, and Beta2) when performing motor attempt tasks under SUD and UD conditions (Figure 6). The differential connectivity maps reveal widespread network topology changes characterized by concurrent significant increases (red) and decreases (blue) in edge strength. This pattern suggests that, when issuing higher-order control intentions, the cortical network of young adults undergoes efficient global reconfiguration and neural resource reallocation via selective strengthening and weakening of connections between specific regions.

#### 3.4.2. Whole-Brain Functional Connectivity Characteristics of Healthy Older Participants

**Figure 7:**
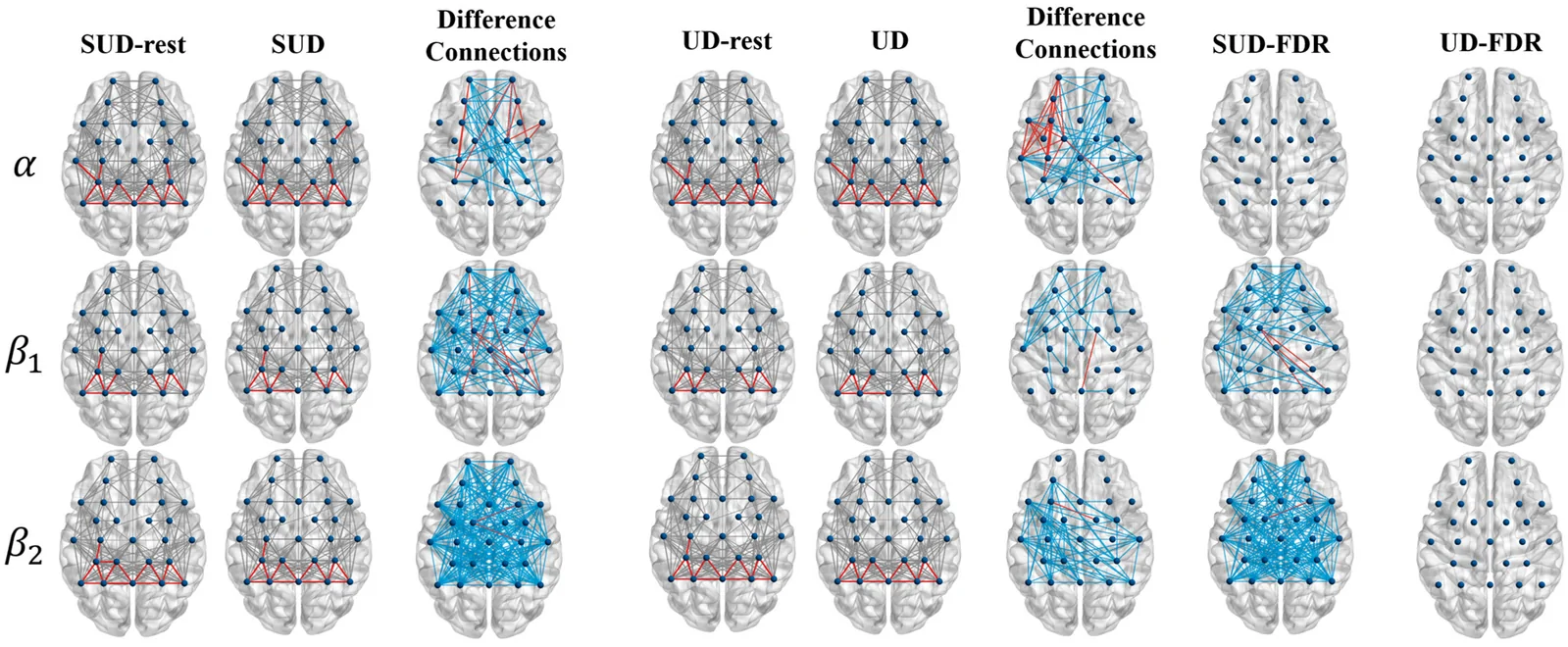
Whole-brain phase-locking value functional connectivity network characteristics during urinary and fecal motor attempt tasks in healthy older participants. Note: SUD—urinary and fecal retention; UD—urination and defecation; α—8–13 Hz; β1—13–20 Hz; β2—20–30 Hz; differential connection locations: red indicates enhancement; blue indicates attenuation.

Healthy older individuals exhibit PLV dynamics during defecation/urination motor tasks that follow overall statistical patterns similar to those of younger adults. During both SUD and UD tasks, whole-brain functional connectivity in the elderly also shows statistically significant differences across all frequency bands (Alpha, Beta1, and Beta2) (Figure 7). The differential networks include complex topological reconfigurations composed of both strengthened (red edges) and weakened (blue edges) functional connections. This suggests that, in healthy aging, cortical regulation of visceral elimination movements preserves widespread and significant whole-frequency-band network coordination and information exchange.

#### 3.4.3. Whole-Brain Functional Connectivity Characteristics in Patients with SCI

**Figure 8:**
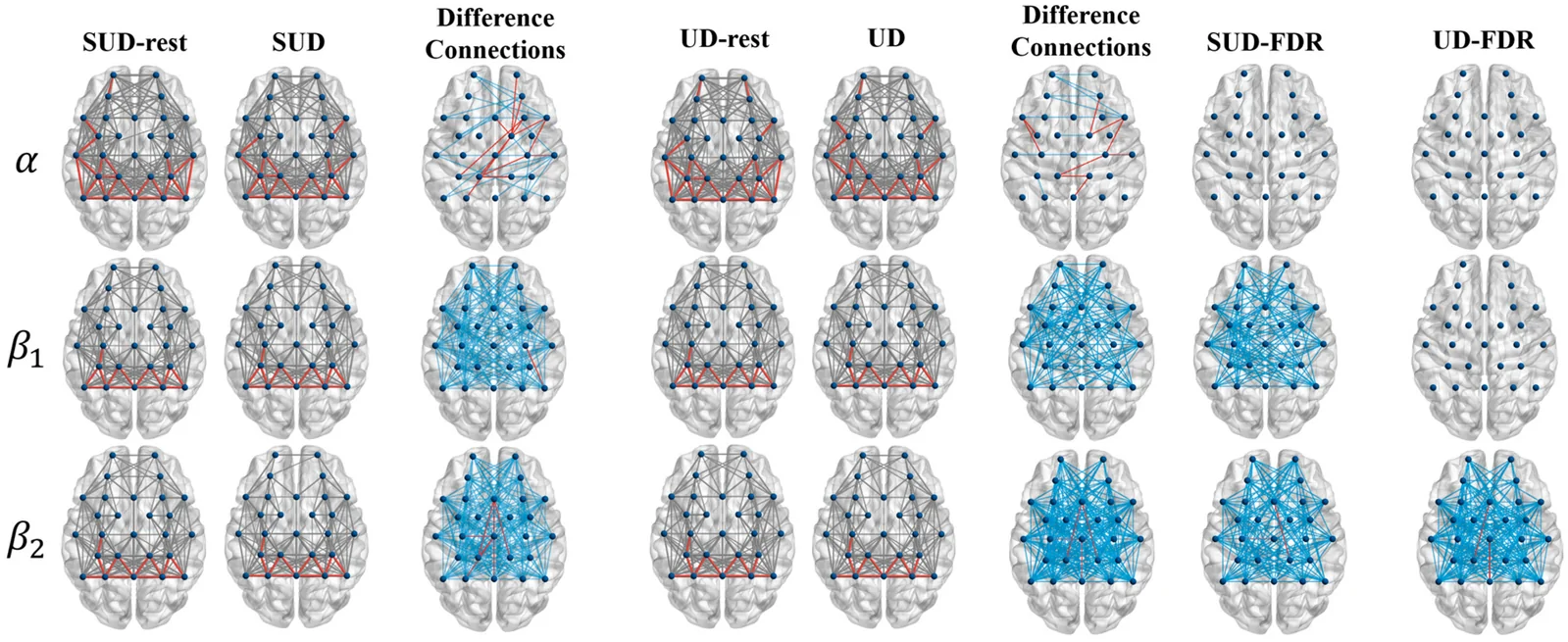
Whole-brain phase-locking value functional connectivity network characteristics in SCI patients during toilet-related motor attempt tasks. Note: SUD—urinary and fecal retention; UD—urination and defecation; α—8–13 Hz; β1—13–20 Hz; β2—20–30 Hz; differential connection locations: red indicates increased connectivity; blue indicates decreased connectivity.

For SCI patients with disruption of descending neural pathways, statistical analysis of whole-brain PLV networks showed distinct pathological manifestations and compensatory features. In the task-evoked activation networks of SCI patients, differential connectivity patterns were likewise observed, predominantly as increases (red; Motor > Rest) and decreases (blue; connectivity reductions) (Figure 8). In a rigorous significance screening of whole-brain network topology in the SCI group, the vast majority of differential edges reached strict statistical significance (*p* < 0.05). However, during the UD task, whole-brain PLV functional connectivity in the Alpha band (8–13 Hz) showed no statistically significant difference in the spinal cord–injured cohort (*p* = 0.13). Except for the Alpha band under the UD task, SCI patients exhibited statistically significant differences in the remaining high-frequency bands (Beta1 and Beta2) during the UD task and across all analyzed frequency bands during the SUD task.

The corrected results confirmed that many connections in the dense uncorrected networks did not survive correction for multiple comparisons. After BH-FDR correction, significant changes were primarily observed in the β bands in the older adult and SCI groups and were predominantly characterized by decreased PLV. No connections in the α band survived FDR correction in any group or task. In the young adult group, only one significantly decreased connection remained in the 13–20 Hz band during the S-UD task. This specificity—the absence of a statistical difference in the UD-task Alpha band—may reflect that, in SCI patients performing active voiding (a maneuver requiring disruption of the preexisting sphincter force equilibrium), cortical low-frequency (Alpha-band) network coordination undergoes pathological decoupling; the central nervous system, in turn, may involve functional compensation of intention transmission by preserving or reinforcing other higher-frequency bands (Beta1 and Beta2) and by maintaining significant network differences under the defensive SUD task.

## 4. Discussion

This study established a standardized dual-visceral electroencephalographic paradigm applicable to both central nervous system-injured patients and healthy individuals, and systematically acquired and quantitatively compared multichannel scalp EEG signals during SUD and UD motor attempts. Through large-scale analyses of EEG topographic features, ERSP, and whole-brain functional connectivity networks in three prototypical cohorts—healthy young adults, healthy older adults, and patients with spinal cord injury—this work provides support for the “cortical exhibits EEG features hypothesis of bowel-and-bladder control intent” of higher-order control intentions for visceral functions, and elucidates, at a deeper level, the spatiotemporal and frequency-specific patterns of coordinated reorganization and compensatory evolution underlying complex visceral motor regulation. Previous EEG studies have provided initial evidence that bladder states and voiding intention are reflected in cortical activity. Colombo et al. [18] reported reduced alpha power during micturition or attempted micturition, which is consistent with the broadband SMC ERD observed in our pelvic motor-attempt tasks. More recently, Zhou et al. showed that urinary urgency can be classified from 64-channel EEG using a deep learning model [20], and further demonstrated the feasibility of wearable single-channel frontal EEG for detecting urgency and voiding intention [19]. These studies support the potential of EEG-based bladder monitoring, but they were conducted mainly in healthy young participants and focused on urinary urgency/voiding rather than combined bowel–bladder attempts. Our study extends this work by characterizing ERSP and PLV features across healthy aging and SCI and by identifying SMC and frontal beta2 as candidate features for future decoding, while also highlighting the need to separate urinary, defecatory, and pelvic-floor components.

The time–frequency evolution and whole-brain topographic analyses in this study first indicate that the cerebral cortex exhibits highly refined frequency-specific and spatially selective cooperative features during the execution of complex visceral motor control for double-voiding. Following the task visual cue, the low-to-mid frequency bands (Alpha and Beta1) in the SMC elicited widespread and reliable ERD. This ubiquitous negative-energy topography was highly concentrated along the midline and bilateral central–parietal regions. According to classical neurophysiological models, ERD in the Alpha and Beta1 bands typically reflects increased local excitability of sensorimotor cortex, the issuance of primary motor intent, and real-time interactions between skeletal muscle and proprioception [32]. The SMC exhibited a broadband, global desynchronization spanning low-to-high frequencies without marked band differentiation, a prototypical multichannel cooperative signature of the human brain issuing strong, direct, and sustained motor intent to peripheral effectors, thereby providing foundational cortical dynamics for the initiation of double-voiding actions.

However, when the analyzed frequency bands shifted toward higher frequencies into the Beta2 band, the whole-brain energy topography underwent a significant frequency-dependent functional reorganization, and electrophysiological features of the frontal cortex began to emerge prominently, manifesting a pronounced “band-splitting” heterogeneous mobilization. Statistical analyses indicated that in the prefrontal region, lower-frequency bands largely exhibited modest ERS elevations or remained near baseline, whereas higher-frequency bands showed a significant shift toward negative ERD. As the central hub for human higher-order cognitive control, volitional inhibition, attentional allocation, and impulse regulation [33], the prefrontal cortex’s selective activation in high-frequency bands may suggest that dual-control regulation is not a superficial, localized initiation of motor actions in a single brain region, but instead depends heavily on precise long-range coordination between parietal sensorimotor networks and frontal higher-order cognitive monitoring networks. In tasks such as SUD that require substantial volitional engagement to counter peripheral reflexes, sustained engagement of frontal high-frequency activity may play a role in maintaining stable cognitive intervention, focusing intention, and actively volitionally suppressing subordinate excretory/reflex centers.

This study, through cross-population comparisons, describes the trajectories of healthy aging and the profound cortical neurophysiological remodeling induced by interruption of central conduction pathways. In the SUD task, group-level statistical differences were confined to high-frequency bands within the SMC. Post hoc multiple comparisons showed that healthy older adults exhibited significantly greater ERD amplitudes in the high-frequency range compared with healthy young adults, whereas SCI showed a similar but non-significant trend. This quantitative pattern aligns with a neurorehabilitation compensation hypothesis: age-related physiological decline in pelvic floor sphincter and neuromuscular control capacity [34,35] or physical interruption of descending neural pathways due to spinal cord injury substantially impairs the effector execution and feedback mechanisms [36,37]. Under these conditions, to overcome a physical conduction block and attempt motor commands, or to sustain high-intensity volitional inhibitory responses during the SUD task, the cerebral cortex appears to engage in active “overshoot” recovery and upward mobilization of resources, prospectively recruiting additional high-frequency, higher-order cognitive and inhibitory-control resources to enforce functional compensation [38].

The intrafrontal frequency-band differentiation further supports this view, although the strength of evidence differs by group and task. During UD, healthy older adults and SCI patients showed significant frontal band differentiation after FDR correction. In contrast, the SCI SUD-FC band effect did not survive correction and should be regarded as an exploratory trend. Healthy young adults did not show significant intrafrontal band differences across task conditions. Quantitatively, these findings indicate that continence control in healthy young individuals is dependent on the automated operation of the SMC somatotopic network, with negligible volitional compensatory load on the frontal cortex; by contrast, older adults and spinal cord-injured patients must heavily recruit high-frequency prefrontal cortical resources to accomplish volitional central reorganization.

The spatial topological characteristics of large-scale whole-brain functional connectivity networks provide a physiological basis for this compensatory mechanism from the perspective of graph theory and global network interactions underlying functional binding. Network analyses indicate that, in healthy elderly subjects during attempts at movement, the strengthened edges of the whole-brain network are markedly biased toward long-range spatial connections between bilateral prefrontal regions and the central sensorimotor area of the prefrontal cortex, reflecting strong long-range monitoring and information integration by control networks over executive networks. As a severe pathological exemplar of complete disruption of descending pathways, SCI patients—while in a movement-attempt state characterized by peripheral complete paralysis and absence of any actual motor output—nevertheless exhibit large-scale, widespread, significantly enhanced synchronized responses in cortical functional networks that closely resemble those of healthy controls. The preserved organization of this whole-brain functional cooperative network provides evidence suggesting that high-level “top-level control intentions” regulating human continence remain distributed across large-scale cortical networks and do not shift or atrophy as a consequence of interrupted peripheral pathways. This furnishes the preliminary brain-network evidence at the electrophysiological level to support noninvasive BCI intention decoding and targeted neuromodulatory rehabilitation training for patients with continence disorders.

This study’s multi-subject experimental data support that, even under extreme pathological conditions such as complete disruption of spinal conductive pathways or focal cortical lesions, the cortical regulation of bowel and bladder functions—encompassing high-level motor intentions and large-scale functional cooperation networks—remains preserved. The elicited broad-band ERD in the SMC, together with frontal high-frequency functional reorganization, constitutes stable characteristic signatures. These findings provide a preliminary empirical foundation and core spatial feature-selection loci (SMC and FC) for the development, in subsequent future work, of highly generalizable and high-accuracy deep learning multiclass decoding models for bowel and bladder functions.

The present study’s uncorrected whole-brain phase-synchrony changes, together with the FDR-corrected beta-band PLV decreases observed in older adults and SCI patients, suggest that large-scale cortical networks undergo task-related reconfiguration. However, the proposed Hebbian interpretation remains speculative and requires validation with simultaneous EOG/EMG and source-level analysis. Delivering intent-driven, top-down cortical commands within this specific temporal window, while concurrently providing peripheral somatosensory afferent feedback, may help recreate at the sacral spinal reflex arc a temporally precise coincidence of presynaptic and postsynaptic discharge. Such coincidence activates activity-dependent neuronal remodeling mechanisms, which in principle may bypass the site of physical injury and restore autonomic control of visceromotor effectors in the pelvic region. This framework provides a neuroscientific theoretical foundation for the present study’s goal of overcoming clinical functional deficits and advancing targeted, noninvasive reparative translation for bowel and bladder dysfunction.

A central limitation of the present study is that both the SUD and UD paradigms combined urination and defecation into single tasks. Specifically, the SUD task required participants to suppress both urination and defecation simultaneously, whereas the UD task required them to attempt both urination and defecation together. As a result, the current experiment cannot determine whether the reported EEG patterns represent urinary intent, defecatory intent, pelvic-floor contraction or relaxation, general motor effort, or a combination of these processes. The SCI cohort was clinically heterogeneous, including AIS grades A–D, variable lesion levels, unrestricted disease duration, and different etiologies. These factors may influence residual sensation, pelvic control, and cortical reorganization. Although we provide exploratory subgroup topographies, the present study was not powered to statistically disentangle these effects. Future multi-center studies with larger, well-stratified SCI cohorts are needed to determine how lesion completeness, chronicity, and bladder/bowel phenotype modulate the EEG signatures reported here. A further limitation is that the frontal ROI (Fp1, Fp2, AF3, AF4) is highly susceptible to ocular and facial-muscle artifacts, particularly in the beta2 band (20–30 Hz). Although extended ICA was applied to remove ocular components, simultaneous EOG/EMG was not recorded, and we cannot fully exclude residual muscular contamination. The frontal beta2 findings should therefore be regarded as exploratory and hypothesis generating rather than as definitive evidence of cortical compensation. Future studies with simultaneous EOG/EMG recording, source-level analysis, and stricter artifact-control procedures are needed to validate these observations.

## 5. Conclusions

This study systematically characterized the spatiotemporal and frequency-specific EEG dynamics underlying bowel and bladder motor attempts in healthy individuals and patients with SCI. Our findings demonstrate that while the sensorimotor cortex provides the foundational broadband ERD for visceral motor initiation, the frontal cortex may play a compensatory role through high-frequency (Beta2) activity, but this interpretation is limited by the lack of significant SCI–young differences and by potential residual artifacts. The evidence for frontal compensation is preliminary and hypothesis generating. The preservation of large-scale, robust cortical networks for continence intent in SCI patients provides preliminary support for the statement of “cortical exhibits EEG features hypothesis of bowel-and-bladder control intent”. Ultimately, these distinct electrophysiological signatures and large-scale topological reconfigurations provide a neuroscientific basis and potential spatial feature-selection targets (SMC and frontal cortex) for developing noninvasive, future BCI decoding models. This work may inform the way for future targeted, closed-loop neurorehabilitation strategies to restore autonomous continence control in patients with neurogenic lower urinary tract and bowel dysfunction.

## Figures and Tables

**Table 1:** Comparison of ERSP Characteristics between participants.

Task—Cortical Regions	Bands	Healthy Young Adults (*n* = 30)	Healthy Older Adults (*n* = 31)	Spinal Cord Injury (*n* = 29)	F	*p*	q
SUD-SMC	α	−0.120 ± 0.615	−0.129 ± 0.557	−0.157 ± 0.853	0.027	0.973	0.973
	β1	−0.120 ± 0.318	−0.222 ± 0.340	−0.266 ± 0.388	1.508	0.226	0.640
	β2	−0.060 ± 0.125	−0.153 ± 0.169	−0.154 ± 0.213	3.452	0.035 *	Na
SUD-FC	α	0.216 ± 0.880	−0.016 ± 0.574	0.304 ± 1.000	1.471	0.235	0.640
	β1	0.157 ± 0.673	−0.032 ± 0.283	−0.008 ± 0.313	1.785	0.173	0.640
	β2	−0.098 ± 0.150	−0.176 ± 0.267	−0.150 ± 0.310	0.908	0.407	0.640
UD-SMC	α	−0.146 ± 0.498	−0.092 ± 0.517	−0.276 ± 0.664	0.957	0.387	0.640
	β1	−0.174 ± 0.337	−0.169 ± 0.339	−0.227 ± 0.294	0.308	0.735	0.898
	β2	−0.108 ± 0.157	−0.117 ± 0.257	−0.135 ± 0.203	0.133	0.875	0.963
UD-FC	α	0.021 ± 0.660	0.250 ± 0.661	0.211 ± 0.666	1.203	0.305	0.640
	β1	−0.013 ± 0.310	0.083 ± 0.270	−0.079 ± 0.254	1.134	0.327	0.640
	β2	−0.137 ± 0.197	−0.175 ± 0.319	−0.183 ± 0.290	0.352	0.705	0.898

SMC—sensorimotor cortex; FC—frontal cortex; SUD—urinary and fecal retention; UD—urination and defecation; α—8–13 Hz; β1—13–20 Hz; β2—20–30 Hz; * denotes *p* < 0.05. Negative ERSP values indicate event-related desynchronization (ERD), and positive values indicate event-related synchronization (ERS). SUD-SMC-beta2 was the pre-specified primary comparison; planned contrasts were Holm–Bonferroni adjusted. All other ERSP comparisons are exploratory and were BH-FDR adjusted across the remaining 11 task × ROI × band tests.

**Table 2:** Comparison of ERSP Characteristics Across Frequency Bands.

Participants	Task	Cortical Regions	α	β1	β2	F	*p*	q
healthy young adults	SUD	SMC	−0.120 ± 0.615	−0.120 ± 0.318	−0.060 ± 0.125	0.248	0.780	0.780
		FC	0.216 ± 0.880	0.157 ± 0.673	−0.098 ± 0.150	2.264	0.109	0.327
	UD	SMC	−0.146 ± 0.498	−0.174 ± 0.337	−0.108 ± 0.157	0.295	0.745	0.780
		FC	0.021 ± 0.660	−0.013 ± 0.310	−0.137 ± 0.197	1.238	0.294	0.588
healthy older adults	SUD	SMC	−0.129 ± 0.557	−0.222 ± 0.340	−0.153 ± 0.169	0.611	0.544	0.780
		FC	−0.016 ± 0.574	−0.032 ± 0.283	−0.176 ± 0.267	1.924	0.151	0.362
	UD	SMC	−0.092 ± 0.517	−0.169 ± 0.339	−0.117 ± 0.257	0.414	0.662	0.780
		FC	0.250 ± 0.661	0.083 ± 0.270	−0.175 ± 0.319	9.004	<0.001	0.0024 *
spinal cord injury	SUD	SMC	−0.157 ± 0.853	−0.266 ± 0.388	−0.154 ± 0.213	0.391	0.677	0.780
		FC	0.304 ± 1.000	−0.008 ± 0.313	−0.150 ± 0.310	4.072	0.020	0.080
	UD	SMC	−0.276 ± 0.664	−0.227 ± 0.294	−0.135 ± 0.203	0.812	0.447	0.766
		FC	0.211 ± 0.666	−0.079 ± 0.303	−0.261 ± 0.366	7.609	<0.001	0.0054 *

SMC denotes the sensorimotor cortex; FC denotes the frontal cortex; SUD—urine and fecal withholding; UD—urination and defecation; α—8–13 Hz; β1—13–20 Hz; β2—20–30 Hz. Negative ERSP values represent event-related desynchronization (ERD), and positive values represent event-related synchronization (ERS). Within-region band effects were compared using one-way ANOVA and corrected across the 12 group × task × ROI tests using Benjamini–Hochberg FDR. * indicates q < 0.05 after FDR correction.

## Data Availability

The data presented in this study are not publicly available due to privacy protection and ethical restrictions related to human EEG recordings from clinical patients and healthy volunteers. The processed data, experimental materials, complete analysis code, and anonymized feature matrices (including ERSP and PLV data) may be made available from the corresponding author upon reasonable request and with permission from the relevant participating institutions.

## Supplementary Materials

The following supporting information can be downloaded at https://www.mdpi.com/article/10.3390/brainsci16101126/s1, Video S1: Real-world setting of the EEG signal acquisition; Video S2: suppressed urination and defecation motor attempt; Video S3: urination and defecation motor attempt; Supplementary Table S1. SCI Clinical characteristics; Supplementary Figure S1. Topographical electroencephalograms of spinal cord injury patients stratified by time since injury. Note: SUD—Suppressed Urination and Defecation; UD—Urination and Defecation; m—month; y—year. Negative ERSP values indicate event-related desynchronization (ERD; blue regions in the topographies), positive values indicate event-related synchronization (ERS; red regions in the topographies), and values near zero indicate no significant change in regional power (yellow–green regions in the topographies). The top three rows show resting-state EEG topographies; the bottom three rows show EEG topographies during the bowel/bladder movement attempt tasks. These topographies are exploratory and descriptive only. No statistical inference was performed due to small and unbalanced subgroup sizes. They are presented to illustrate potential trends and to generate hypotheses for future studies; Supplementary Figure S2. Topographical electroencephalograms of spinal cord injury patients stratified by ASIA. Note: SUD—Suppressed Urination and Defecation; UD—Urination and Defecation; A—ASIA A; B—ASIA B; C—ASIA C; D—ASIA D. Negative ERSP values indicate event-related desynchronization (ERD; blue regions in the topographies), positive values indicate event-related synchronization (ERS; red regions in the topographies), and values near zero indicate no significant change in regional power (yellow–green regions in the topographies). The top three rows show resting-state EEG topographies; the bottom three rows show EEG topographies during the bowel/bladder movement attempt tasks. These topographies are exploratory and descriptive only. No statistical inference was performed due to small and unbalanced subgroup sizes. They are presented to illustrate potential trends and to generate hypotheses for future studies; Supplementary Figure S3. Topographical electroencephalograms of spinal cord injury patients stratified by pelvic sensory and motor. Note: SUD—Suppressed Urination and Defecation; UD—Urination and Defecation; m—motor; s—sensory. Negative ERSP values indicate event-related desynchronization (ERD; blue regions in the topographies), positive values indicate event-related synchronization (ERS; red regions in the topographies), and values near zero indicate no significant change in regional power (yellow–green regions in the topographies). The top three rows show resting-state EEG topographies; the bottom three rows show EEG topographies during the bowel/bladder movement attempt tasks. These topographies are exploratory and descriptive only. No statistical inference was performed due to small and unbalanced subgroup sizes. They are presented to illustrate potential trends and to generate hypotheses for future studies; Supplementary Figure S4. Topographical electroencephalograms of spinal cord injury patients stratified by etiology. Note: SUD—Suppressed Urination and Defecation; UD—Urination and Defecation; My—myelitis; Tr—trauma; Tu—tumor. Negative ERSP values indicate event-related desynchronization (ERD; blue regions in the topographies), positive values indicate event-related synchronization (ERS; red regions in the topographies), and values near zero indicate no significant change in regional power (yellow–green regions in the topographies). The top three rows show resting-state EEG topographies; the bottom three rows show EEG topographies during the bowel/bladder movement attempt tasks. These topographies are exploratory and descriptive only. No statistical inference was performed due to small and unbalanced subgroup sizes. They are presented to illustrate potential trends and to generate hypotheses for future studies.

## Abbreviations

The following abbreviations are used in this manuscript:

ANOVA	Analysis of Variance
ASIA	American Spinal Injury Association
BCI	Brain–Computer Interface
CAR	Common Average Reference
EEG	Electroencephalography/Electroencephalographic
ERD	Event-Related Desynchronization
ERP	Event-Related Potential
ERS	Event-Related Synchronization
ERSP	Event-Related Spectral Perturbation
FC	Frontal Cortex
ICA	Independent Component Analysis
MA	Motor Attempt
MI	Motor Imagery
NBD	Neurogenic Bowel Dysfunction
NLUTD	Neurogenic Lower Urinary Tract Dysfunction
PLV	Phase-Locking Value
ROI	Region of Interest
SCI	Spinal Cord Injury
SMC	Sensorimotor Cortex
SUD	Suppressed Urination and Defecation
UD	Urination and Defecation
