# Supplementary material for: EEG Features of Cortical Intent for Urination and Defecation in Healthy Aging and Spinal Cord Injury: A Descriptive Feasibility Study for BCI Neurorehabilitation
Source: Brain Sci. 2026 Oct 18;16(10):1126. doi: 10.3390/brainsci16101126 (PMC13702114; doi:10.3390/brainsci16101126)
Supplement: Supplementary file 1 [file brainsci-16-01126-s001.zip › Supplementary Figures S1-4 and Table S1.pdf]

Supplementary Table S1. SCI Clinical characteristics.

Participants	Sex	Age,years	Etiology	Lesion level	Duration, month	AIS grade	Pelvic sensory	Pelvic motor
#1	Female	47	Myelitis	C5	3	A	0	0
#2	male	19	Trauma	L5	1	D	1	1
#3	Female	39	Myelitis	C1	2	D	1	1
#4	Female	76	Tumor	T5	180	C	1	1
#5	male	65	Trauma	T8	23	A	0	0
#6	male	53	Trauma	T4	28	B	1	0
#7	male	65	Trauma	C6	22	A	0	0
#8	Female	58	Myelitis	L3	1	C	1	1
#9	Female	72	Trauma	T5	4	B	1	0
#10	Female	45	Trauma	T12	12	D	1	1
#11	Female	18	Trauma	L3	5	D	1	1
#12	male	56	Trauma	T11	240	B	1	0
#13	male	34	Trauma	T4	12	D	1	1
#14	male	54	Trauma	C4	6	A	0	0
#15	Female	26	Trauma	T5	2	C	1	1
#16	male	43	Trauma	C2	9	A	0	0
#17	Female	27	Trauma	T1	5	B	1	0
#18	male	29	Myelitis	C3	2	D	1	1
#19	male	61	Tumor	L2	4	B	1	0
#20	male	54	Trauma	C3	5	D	1	1
#21	Female	72	Tumor	T8	1	B	1	0
#22	male	47	Trauma	L5	5	D	1	1
#23	male	26	Trauma	C2	27	D	1	1
#24	Female	49	Tumor	C7	384	C	1	0
#25	Female	47	Trauma	T6	336	A	0	0
#26	Female	33	Trauma	T3	120	D	1	1
#27	male	48	Trauma	T12	264	D	1	1
#28	Female	47	Trauma	L1	348	D	1	1
#29	male	48	Trauma	C2	10	D	1	1

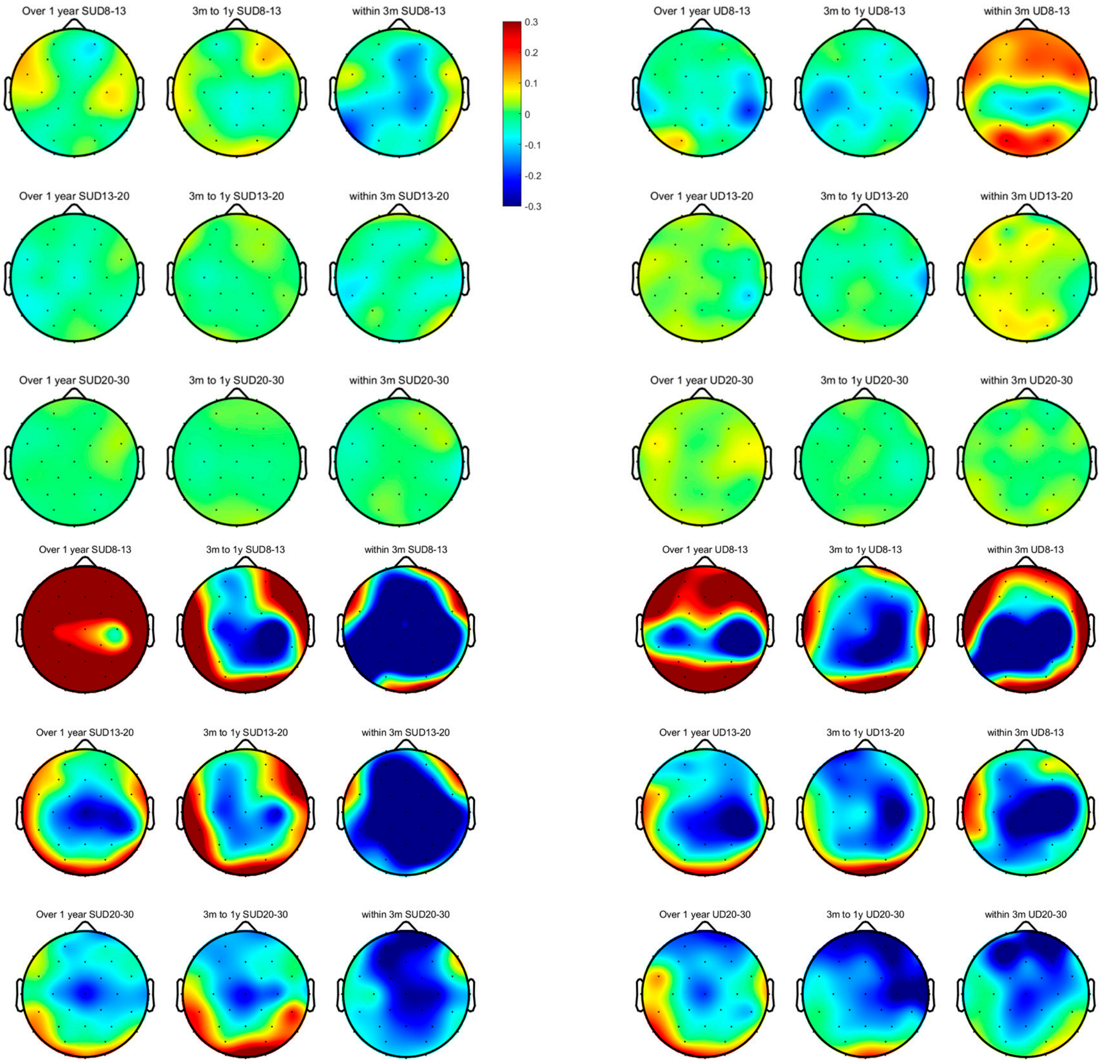

Supplementary Figure S1. Topographical electroencephalograms of spinal cord injury patients stratified by time since injury. Note: SUD—Suppressed Urination and Defecation; UD—Urination and Defecation; m—month; y—year. Negative ERSP values indicate event-related desynchronization (ERD; blue regions in the topographies), positive values indicate event-related synchronization (ERS; red regions in the topographies), and values near zero indicate no significant change in regional power (yellow–green regions in the topographies). The top three rows show resting-state EEG topographies; the bottom three rows show EEG topogra-phies during the bowel/bladder movement attempt tasks. These topographies are exploratory and descriptive only. No statistical inference was performed due to small and unbalanced subgroup sizes. They are presented to illustrate potential trends and to generate hypotheses for future studies.

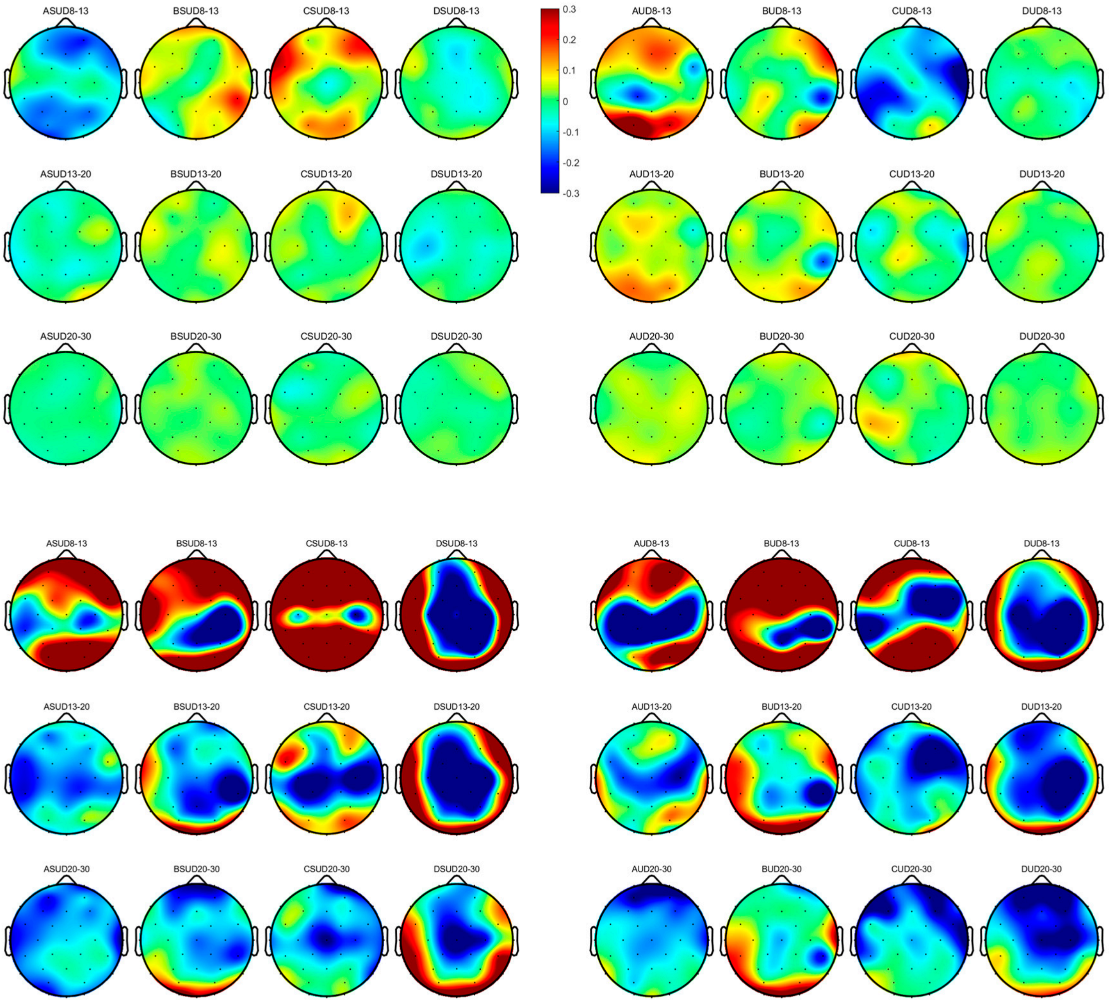

Supplementary Figure S2. Topographical electroencephalograms of spinal cord injury patients stratified by ASIA. Note: SUD—Suppressed Urination and Defecation; UD—Urination and Defecation; A—ASIA A; B—ASIA B; C—ASIA C; D—ASIA D. Negative ERSP values indicate event-related desynchronization (ERD; blue regions in the topographies), positive values indicate event-related synchronization (ERS; red regions in the topographies), and values near zero indicate no significant change in regional power (yellow–green regions in the topographies). The top three rows show resting-state EEG topographies; the bottom three rows show EEG topogra-phies during the bowel/bladder movement attempt tasks. These topographies are exploratory and descriptive only. No statistical inference was performed due to small and unbalanced subgroup sizes. They are presented to illustrate potential trends and to generate hypotheses for future studies.

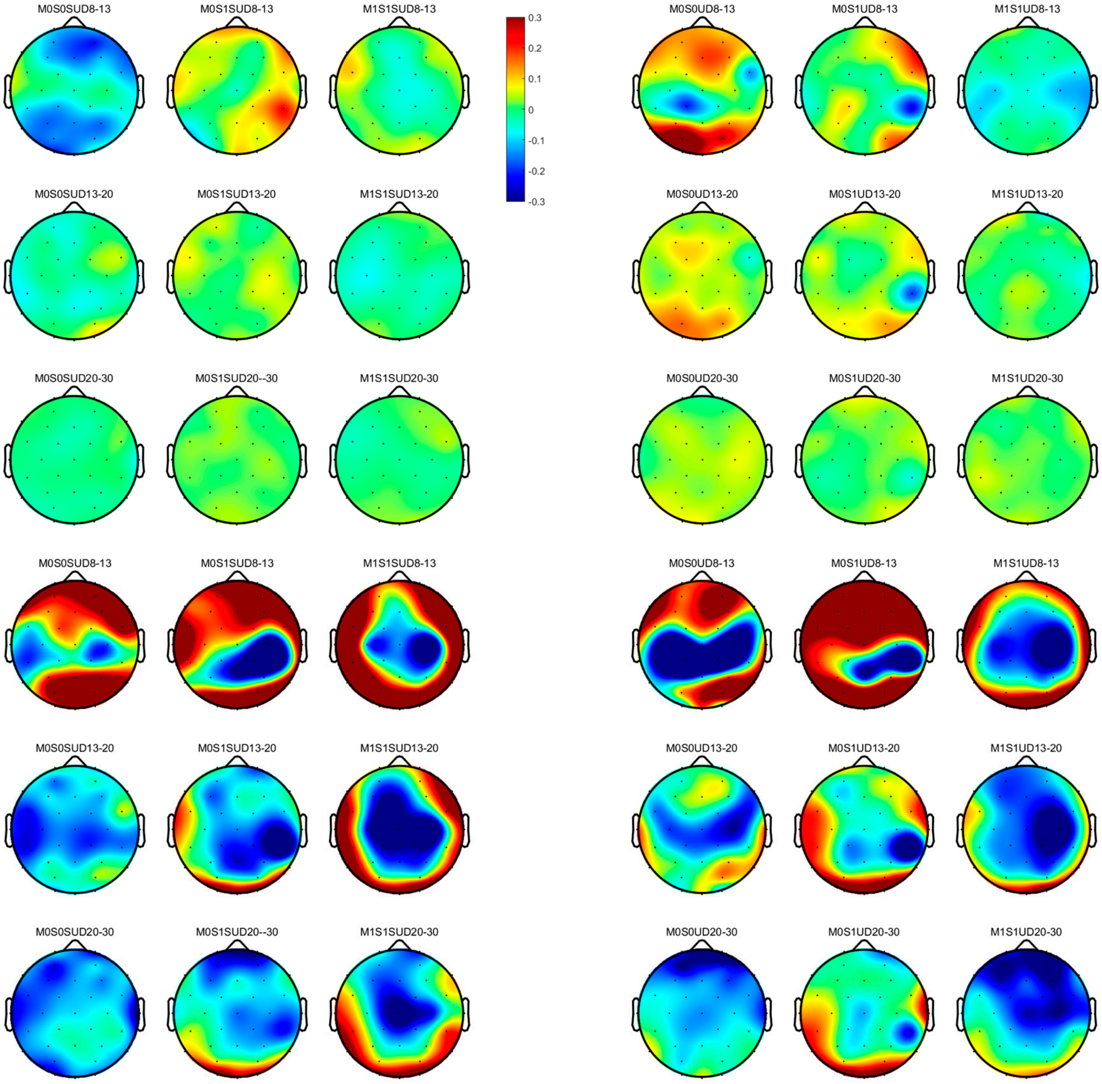

Supplementary Figure S3. Topographical electroencephalograms of spinal cord injury patients stratified by pelvic sensory and motor. Note: SUD—Suppressed Urination and Defecation; UD—Urination and Defecation; m—motor; s—sensory. Negative ERSP values indicate event-related desynchronization (ERD; blue regions in the topographies), positive values indicate event-related synchronization (ERS; red regions in the topographies), and values near zero indicate no significant change in regional power (yellow–green regions in the topographies). The top three rows show resting-state EEG topographies; the bottom three rows show EEG topogra-phies during the bowel/bladder movement attempt tasks. These topographies are exploratory and descriptive only. No statistical inference was performed due to small and unbalanced subgroup sizes. They are presented to illustrate potential trends and to generate hypotheses for future studies.

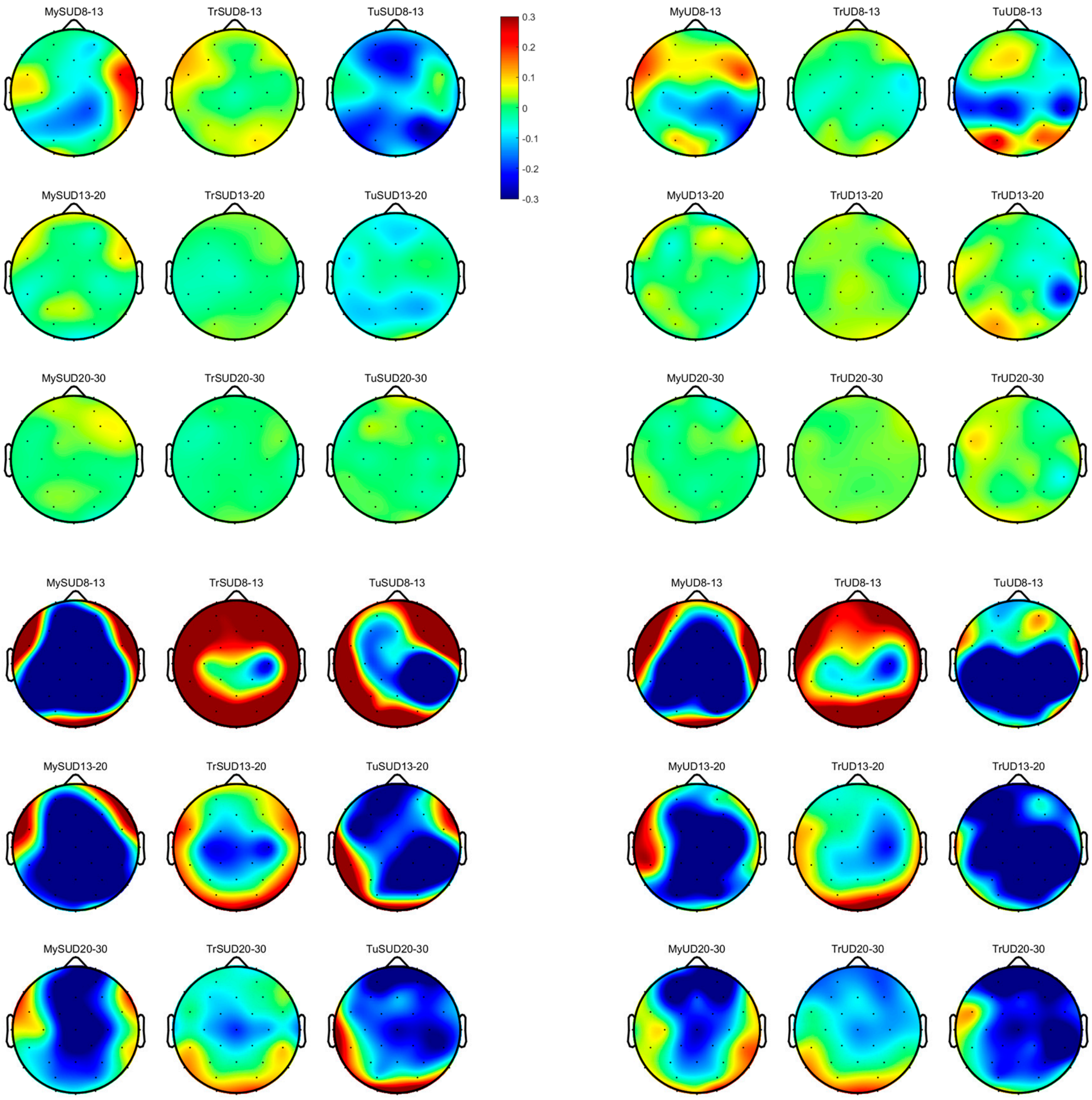

Supplementary Figure S4. Topographical electroencephalograms of spinal cord injury patients stratified by etiology. Note: SUD—Suppressed Urination and Defecation; UD —Urination and Defecation; My—myelitis; Tr—trauma; Tu—tumor. Negative ERSP values indicate event-related desynchronization (ERD; blue regions in the topographies), positive values indicate event-related synchronization (ERS; red regions in the topographies), and values near zero indicate no significant change in regional power (yellow–green regions in the topographies). The top three rows show resting-state EEG topographies; the bottom three rows show EEG topogra-phies during the bowel/bladder movement attempt tasks. These topographies are exploratory and descriptive only. No statistical inference was performed due to small and unbalanced subgroup sizes. They are presented to illustrate potential trends and to generate hypotheses for future studies.
